# Development-Dependent Plasticity in Vasoactive Intestinal Polypeptide Neurons in the Infralimbic Cortex

**DOI:** 10.1093/texcom/tgab007

**Published:** 2021-02-04

**Authors:** Stuart A Collins, Ipe Ninan

**Affiliations:** Department of Neurosciences, University of Toledo, Toledo, OH 43614, USA

**Keywords:** adolescence, GABA, infralimbic cortex, synaptic plasticity, VIP neurons

## Abstract

The onset of several neuropsychiatric disorders including anxiety disorders coincides with adolescence. Consistently, threat extinction, which plays a key role in the regulation of anxiety-related behaviors, is diminished during adolescence. Furthermore, this attenuated threat extinction during adolescence is associated with an altered synaptic plasticity in the infralimbic medial prefrontal cortex (IL-mPFC), a brain region critical for threat extinction. However, the mechanism underlying the altered plasticity in the IL-mPFC during adolescence is unclear. Given the purported role of vasoactive intestinal polypeptide expressing interneurons (VIPINs) in disinhibition and hence their potential to affect cortical plasticity, we examined whether VIPINs exhibit an adolescence-specific plasticity in the IL-mPFC. We observed an increase in GABAergic transmission and a decrease in excitability in VIPINs during adolescence. Male mice show a significantly higher VIPIN-pyramidal neuron GABAergic transmission compared with female mice. The observed increase in GABAergic transmission and a decrease in membrane excitability in VIPINs during adolescence could play a role in the altered plasticity in the adolescent IL-mPFC. Furthermore, the suppression of VIPIN-mediated GABAergic transmission in females might be relevant to sex differences in anxiety disorders.

## Introduction

Adolescence is a critical development period during which the prefrontal cortex undergoes a prolonged rearrangement (Insel 2010; Cohen et al. 2016). The notion that phylogenetically newer brain areas undergo a protracted development is supported by recent studies demonstrating the slow maturation of GABAergic activity in the medial prefrontal cortex (mPFC) (Wang and Gao 2010; Yang et al. 2014; Pan et al. 2016; Miyamae et al. 2017; Koppensteiner et al. 2019c). This delayed development of mPFC GABAergic activity involves a complex and cell type-specific maturation of membrane and synaptic properties (Koppensteiner et al. 2019c). It is plausible that these developmental events could interact with both genetic and environmental risk factors leading to neuropsychiatric disorders, particularly anxiety disorders and schizophrenia (Loranger 1984; Pollack et al. 1996; Kim-Cohen et al. 2003; Dalsgaard et al. 2019). Therefore, an in-depth understanding of the development of the mPFC from preadolescence to adulthood might provide important insights into the developmental mechanisms underlying these disorders.

Congruent with an increase in anxiety disorders during adolescence, threat extinction, which is believed to play a critical role in regulating anxiety behaviors, is suppressed during adolescence (McCallum et al. 2010; Pattwell et al. 2012; Koppensteiner et al. 2019c; Ressler 2020). This diminished threat extinction during adolescence is associated with an altered synaptic and intrinsic plasticity in the infralimbic medial prefrontal cortex (IL-mPFC) pyramidal neurons (Pattwell et al. 2012; Koppensteiner et al. 2019a). The IL-mPFC plays a critical role in the expression of threat extinction (Milad and Quirk 2002). Consistently, the IL-mPFC is believed to regulate the visceromotor and autonomic functions (Vertes 2004). Since the synaptic inhibition plays an important role in the regulation of plasticity in glutamatergic neurons (Couey et al. 2007; Sakata et al. 2009), we have recently undertaken experiments to examine the changes in both the synaptic and intrinsic properties of the IL-mPFC GABAergic neurons during the transitions into and out of adolescence. Among the 3 major classes of GABAergic neurons, parvalbumin-expressing (PVINs), somatostatin-expressing (SSTINs), and the 5-HT3a receptor-expressing interneurons, the SSTINs exhibit an elevated inhibition of pyramidal neurons in the IL-mPFC during adolescence, while PVIN-mediated inhibition of pyramidal neurons undergoes a protracted development until adulthood (Koppensteiner et al. 2019c). Given the effect of SSTIN activity on cortical glutamatergic synapses (Urban-Ciecko et al. 2015), it is plausible that the adolescence-specific plasticity mediated by SSTINs could alter cortical plasticity. However, the mechanism underlying this rare plasticity in SSTINs during adolescence is unknown. Since the vasoactive intestinal polypeptide expressing interneurons (VIPINs), a subclass of 5-HT3a receptor-expressing interneurons, are the major upstream modulators of SSTINs (Lee et al. 2013; Pfeffer et al. 2013; Pi et al. 2013; Wamsley and Fishell 2017; Adler et al. 2019), it is important to know whether VIPINs show an adolescence-specific plasticity, as it could affect the output of the downstream SSTINs.

We examined the synaptic and membrane properties of VIPINs in preadolescent, adolescent, and adult mice. Our results show an increased synaptic inhibition of VIPINs during adolescence. Furthermore, VIPIN-pyramidal neuron GABAergic transmission is enhanced in male mice during preadolescence and adolescence, but this sex difference is absent in adult mice. These findings might be relevant to sex differences in anxiety disorders, which are prevalent during adolescence.

## Materials and Methods

### Animals

Female and male preadolescent (postnatal day 24, P24), adolescent (P29), and adult mice (>P60) were used in this study (Koppensteiner et al. 2019b; Koppensteiner et al. 2019c; Spear 2000). VIP-tdTomato mice were generated by crossing Vip-IRES-cre (C57BL/6 J) (Stock #: 031628, The Jackson Laboratory**)** and B6.Cg-Gt(ROSA)26Sortm14(CAG-tdTomato)Hze/J (Stock #: 007914, The Jackson Laboratory) mice. VIP-ChR2 mice were generated by crossing Vip-IRES-cre (C57BL/6 J) and Ai32(RCL-ChR2(H134R)/EYFP) (Stock #: 024109, The Jackson Laboratory) mice. Mice were maintained on a 12-h light–dark cycle at 23 °C with access to food and water ad libitum. The Institutional Animal Care and Use Committee of the University of Toledo approved all the procedures.

### Electrophysiology

A transcardial perfusion was performed in anesthetized mice (pentobarbital 120 mg//kg) for approximately 45 s. With ice-cold oxygenated artificial cerebrospinal fluid (ACSF) containing (in mM): NaCl (118), glucose (10), KCl (2.5), NaH_2_PO_4_ (1), CaCl_2_ (1), and MgSO_4_ (1.5) (325 mOsm, pH 7.4). Immediately after the perfusion, brains were isolated for preparing 300 μm prefrontal cortical slices on a vibratome (Campden Instruments). Slices were incubated in ACSF at room temperature for a minimum of 1 h followed by the transfer of brain slices to the recording chamber superfused with the aforementioned ACSF containing 2.5 mM CaCl_2_ at 32 °C flowing at 2 mL/min rate. The IL-mPFC was located using a 4x objective. The tdTomato expressing VIPINs were identified using fluorescence microscopy and the recorded neurons were visualized using a 40x water immersion objective and video-enhanced differential interference contrast microscopy. Pyramidal neurons were identified by their morphology and accommodating action potential firing characteristics. Recording electrodes of 3–5 MΩ resistance were filled with an internal solution containing (in mM): K-gluconate (130), KCl (10), MgCl_2_ (5), MgATP (5), NaGTP (0.2), EGTA (0.5), HEPES (5), pH adjusted to 7.4 with KOH. Electrophysiological recordings were performed with a Multiclamp 700B amplifier connected to a Digidata 1550A (Molecular Devices, CA, United States of America). Signals were sampled at 20–100 kHz and filtered at 2 kHz. Neuronal excitability was measured in current clamp mode by injecting currents from −50 to 150 pA (10 pA increments). Spontaneous excitatory postsynaptic currents (sEPSCs) were recorded at −60 mV in the presence of bicuculline (10 μM). Spontaneous inhibitory postsynaptic currents (sIPSCs) were recorded at −60 mV in the presence of DNQX (10 μM) and APV (50 μM). In experiments involving electrical stimulation, a concentric bipolar stimulating electrode was placed in layer 5. NMDA excitatory postsynaptic currents (EPSCs) were measured at +40 mV in the presence of bicuculline (10 μM) and DNQX (10 μM) using an electrode solution containing (in mM): CsCl (130), HEPES (10), EGTA (0.5), MgATP (5), NaGTP (0.2), QX314 (5), pH adjusted to 7.4 with CsOH. The CsCl internal solution containing 10 mM spermine was used for rectification index experiments. AMPA EPSCs were evoked at different holding potentials from −60 to +60 mV (20 mV increments) in the presence of bicuculline (10 μM) and APV (50 μM), and rectification index was calculated as the ratio of the slopes of the linear current/voltage relationship at positive (0–60 mV) and negative (−60–0 mV) holding potentials (Adesnik and Nicoll 2007). For light stimulation of GABAergic terminals in VIP-ChR2 mice, blue light (470 nm) was emitted from a Lumen 1600-LED (Prior) at increasing durations (0.25–0.8 ms, 0.1 Hz) in the presence of DNQX (10 μM) and APV (50 μM). Electrophysiological recordings were rejected when series resistance or holding current changed by 10% or more.

### Microscopy

Following euthanasia with pentobarbital (120 mg/kg), brains from VIP-tdTomato mice were fixed in 4% PFA in PBS for 24 h at 4 °C before being placed in 30% sucrose for 24 h at 4 °C. Brains were frozen on dry ice and 40 μm cryosections of the mPFC were mounted on slides and cover-slipped with Fluoromount G. Confocal images of the IL-mPFC were taken from 4 sections for each animal, using a TCS SP5 multiphoton laser-scanning confocal microscope (Leica Microsystems, Buffalo Grove, IL) with a 10x objective lens. Maintaining the same laser intensity, exposure time, camera gain, and neutral density filter settings, Z-stack images (1-μm optical sections) were acquired and 3D projections were constructed with the Leica AFM acquisition/analytical software suite. By using a methodology described earlier (Grider et al. 2006), confocal images were converted to grayscale 8-bit images and feature-extracted for VIP fibers through a Hessian-based filter in NIH ImageJ (version 1.44) software plugin, FeatureJ. FeatureJ-based filtering of images was performed utilizing the smallest eigenvalue of Hessian tensor option (smoothing scale 1). The image was then converted to a binary image via the use of a threshold value determined to accurately depict fibers in test images. A blind observer analyzed the images across all layers of the IL-mPFC and the average intensity was determined through the use of the analyze function in ImageJ. We measured the soma area of VIPINs in the IL-mPFC by applying an automated process in Image J using the same images as those used to measure neurite density. After the scaling was set using the “set scale” feature in ImageJ, the images were converted to 8-bit grayscale and the threshold feature in ImageJ was used to filter out much of the neurite and background fluorescence. This setting was predetermined and the same threshold was utilized throughout the measurements. If it was noticed that cells were overlapping, the area containing those cells was cleared and they were not included in measurements. A box was drawn over the majority of the IL-mPFC and the “analyze particles” program in ImageJ was used to measure soma area. We excluded particles with a size smaller than 40 μm^2^. Soma area measurements from each animal were expressed as an average soma area from all of the VIP neurons measured (87–218 neurons per animal) in the 4 IL-mPFC images from each animal.

### Data analysis

Membrane properties and evoked currents were analyzed using Clampfit 10.7. Passive membrane properties were measured from the membrane voltage response to hyperpolarizing current injections of −50 to −10 pA. Input resistance (R_in_) was calculated as the slope of the current–voltage relationship from −50 to 0 pA and the membrane time constant tau was calculated by fitting the initial change in membrane voltage in response to a − 50 pA step to a single exponential function. Membrane capacitance (C_m_) was calculated from tau and R_in_. Spontaneous synaptic transmission was analyzed using MiniAnalysis program (Synaptosoft). A single exponential fit using standard exponential formula was used for measuring NMDA EPSC decay time. Statistical analyses were performed using GraphPad Prism 8 (GraphPad Software, Inc) or IBM SPSS statistics (version 26) software. Two-way ANOVA followed by Bonferroni post hoc test was used for analyzing spontaneous currents, rectification index, NMDA EPSC decay time, VIP tdTomato fluorescence intensity, VIPIN soma area, NMDA EPSCs, inhibitory postsynaptic currents (IPSCs), and membrane properties.

## Results

### Membrane properties of IL-mPFC VIPINs in preadolescent, adolescent, and adult mice

**Figure 1:**
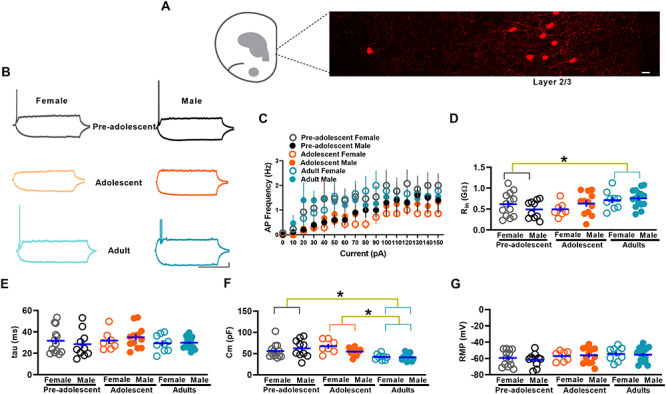
Membrane properties of IL-mPFC VIPINs in preadolescent, adolescent, and adult mice. **A**) Schematic showing the IL-mPFC and an image of tdTomato-expressing VIPINs in the IL-mPFC (from an adult female VIPIN-tdTomato mouse). Scale 10 μm. **B**) Example traces of voltage responses to hyperpolarizing (−30 pA) and depolarizing (+30 pA) current steps in tdTomato-expressing VIPINs. Scale = 500 ms/10 mV. **C**) Mean action potential (AP) frequency in response to current injection in VIPINs from female preadolescent (n = 13 neurons/5 mice), male preadolescent (n = 10 neurons/4 mice), female adolescent (n = 7 neurons/4 mice), male adolescent (n = 12 neurons/4 mice), female adult (n = 9 neurons/3 mice), and male adult mice (n = 15 neurons/5 mice). A two-way ANOVA of action potential frequency showed an effect of age [F (2, 60) = 3.7, *P* = 0.03, *P* = 0.7 for preadolescent vs adolescent, *P* = 0.014 for adult vs adolescent, *P* = 0.22 for adult vs preadolescent] but no effect of sex [F (1, 60) = 0.004, *P* = 0.95] or an interaction between age and sex [F (2, 60) = 1.04, *P* = 0.36]. A two-way ANOVA of input resistance (Rin, **D**) showed an effect of age [F (2, 60) = 4.07, *P* = 0.02, *P* = 1 for preadolescent vs adolescent, *P* = 0.09 for adult vs adolescent, *P* = 0.035 for adult vs preadolescent] but no effect of sex [F (1, 60) = 0.1, *P* = 0.7] or an interaction between age and sex [F (2, 60) = 1.6, *P* = 0.2]. A two-way ANOVA of membrane capacitance (Cm, **F**) showed an effect of age [F (2, 60) = 8, *P* = 0.001, *P* = 0.8 for preadolescent vs adolescent, *P* = 0.001 for adult vs adolescent, *P* = 0.015 for adult vs preadolescent] but no effect of sex [F (1, 60) = 0.04, *P* = 0.8] or an interaction between age and sex [F (2, 60) = 0.28, *P* = 0.75]. A two-way ANOVA of membrane time constant (tau, **E**) did not show an effect of age [F (2, 60) = 0.83, *P* = 0.44], sex [F (1, 60) = 0.001, *P* = 0.97] or an interaction between age and sex [F (2, 60) = 0.53, *P* = 0.59]. A two-way ANOVA of resting membrane potential (RMP, **G**) did not show an effect of age [F (2, 60) = 1.3, *P* = 0.1], sex [F (1, 60) = 0.12, *P* = 0.73] or an interaction between age and sex [F (2, 60) = 0.17, *P* = 0.85].

We have previously demonstrated the differential maturation of membrane properties in PVINs and SSTINs in the IL-mPFC during preadolescence to adulthood (Koppensteiner et al. 2019c). To examine whether the excitability of VIPINs undergoes development-dependent changes from preadolescence to adulthood, we first compared the number of evoked action potentials in response to current injection in preadolescent, adolescent, and adult mice. As described before (Anastasiades et al. 2019), we observed a predominant presence of tdTomato-expressing VIPINs in the superficial layers compared with the deeper layers of the IL-mPFC in VIP-tdTomato mice (Fig. 1*A*). We undertook whole cell recording in tdTomato-expressing layer 2/3 VIPINs. Despite a low number of evoked action potentials in all the groups, we observed an effect of age on the frequency of evoked action potentials and the adolescent group showed a significantly lower action potential frequency compared with adult group (comparison at +30pA) (Fig. 1*B*, *C*). However, we did not observe an effect of sex or an interaction between age and sex. We also observed an increase in input resistance and a decrease in membrane capacitance in the adult group, while both the membrane time constant and resting membrane potential did not change during development from preadolescence to adulthood (Fig. 1*D–G*). A comparison of number of action potentials, input resistance, membrane time constant, membrane capacitance, or resting membrane potentials in female and male groups did not show a statistically significant effect, suggesting a lack of sex difference in membrane properties. Overall, our examination of membrane properties shows an adolescence-specific decrease in membrane excitability in VIPINs.

Given that we observed far fewer evoked action potentials in VIPINs (Fig. 1*B*, *C*) than in other IL-mPFC neurons (Koppensteiner et al. 2019a; Koppensteiner et al. 2019c), we examined whether the excitability in VIPINs is temperature-dependent. A comparison of the number of evoked action potentials in response to current injection in brain slices maintained at 24 °C and 32 °C revealed a dramatic decrease in evoked action potentials in the 32 °C group compared with the 24 °C group (Supplementary Fig. 1*A*, *B*). However, we did not observe an effect of temperature on input resistance, membrane time constant, membrane capacitance, or resting membrane potential (Supplementary Fig. 1*C–F*).

### Spontaneous glutamatergic transmission in IL-mPFC VIPINs in preadolescent, adolescent, and adult mice

**Figure 2:**
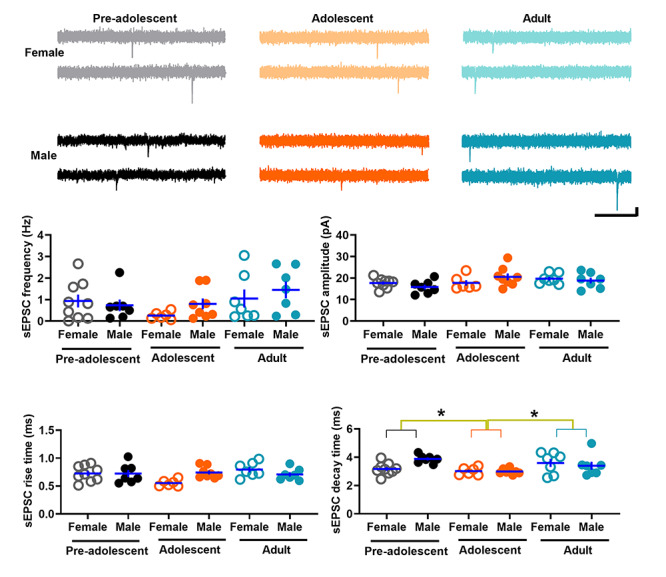
Spontaneous glutamatergic transmission in the IL-mPFC VIPINs of preadolescent, adolescent, and adult mice. Frequency, amplitude, rise time, and decay time of sEPSCs in tdTomato-expressing VIPINs in the IL-mPFC from female preadolescent (n = 9 neurons/3 mice), male preadolescent (n = 7 neurons/3 mice), female adolescent (n = 6 neurons/3 mice), male adolescent (n = 8 neurons/3 mice), female adult (n = 7 neurons/3 mice), and male adult mice (n = 7 neurons/3 mice). Horizontal line in each group represents mean and vertical line represents SEM. Upper panels show example traces. Scale, 250 ms/10 pA. A two-way ANOVA of sEPSC frequency did not show an effect of age [F (2, 43) = 2.5, *P* = 0.9], an effect of sex [F (1, 43) = 0.9, *P* = 0.4] or an interaction between age and sex [F (2, 43) = 0.8, *P* = 0.4]. Similarly, we did not observe an effect of age [F (2, 43) = 2.8, *P* = 0.07], an effect of sex [F (1, 43) = 0.001, *P* = 1] or an interaction between age and sex [F (2, 43) = 2.1, *P* = 0.14] in sEPSC amplitude measurements. Although a two-way ANOVA of sEPSC rise time did not show an effect of age [F (2, 43) = 2.6, *P* = 0.09] or sex [F (1, 43) = 0.8, *P* = 0.4], there was a significant interaction between age and sex [F (2, 43) = 4.5, *P* = 0.018]. We observed an effect of age [F (2, 43) = 4.9, *P* = 0.012, *P* = 0.036 for preadolescent vs adolescent, *P* = 0.037 for adult vs adolescent, *P* = 1 for adult vs preadolescent] and an interaction between age and sex [F (2, 43) = 3.5, *P* = 0.04] in sEPSC decay time measurements. However, we did not observe an effect of sex on sEPSC decay time [F (1, 43) = 1.2, *P* = 0.28].

To understand whether VIPINs exhibit a development-dependent change in glutamatergic synaptic transmission, we compared the frequency, amplitude, rise time, and decay time of sEPSCs in VIPINs from preadolescent, adolescent, and adult groups. We observed a minimal spontaneous glutamatergic transmission in VIPINs from all 3 groups (Fig. 2). The lack of effect of developmental stage on sEPSC frequency and amplitude suggested that the overall strength of glutamatergic synapses does not change during development from preadolescence to adulthood. However, an age-dependent effect on sEPSC decay time and an interaction of age and sex on sEPSC rise and decay kinetics strongly suggest a sex- and development-dependent modulation of the subunit composition of AMPA receptors in VIPINs.

### Evoked glutamatergic transmission in IL-mPFC VIPINs in preadolescent, adolescent, and adult mice

**Figure 3:**
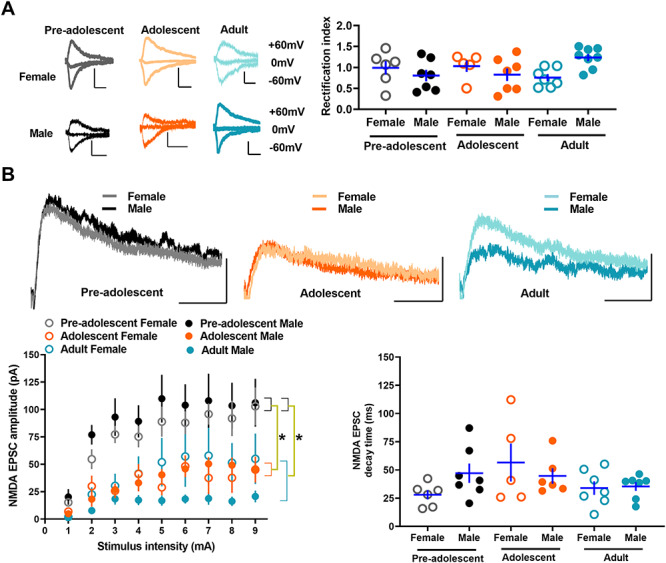
Evoked glutamatergic transmission in the IL-mPFC VIPINs of preadolescent, adolescent, and adult mice. **A**) Synaptic CPARs in IL-mPFC VIPINs. Rectification index in tdTomato-expressing IL-mPFC VIPINs from female preadolescent (n = 6 neurons/3 mice), male preadolescent (n = 7 neurons/3 mice), female adolescent (n = 5 neurons/3 mice), male adolescent (n = 7 neurons/3 mice), female adult (n = 7 neurons/3 mice), and male adult mice (n = 8 neurons/3 mice). Although a two-way ANOVA of rectification index did not show an effect of age [F (2, 39) = 0.3, *P* = 0.7] or an effect of sex [F (1, 39) = 0.08, *P* = 0.8], there was a significant interaction between age and sex [F (2, 39) = 4.9, *P* = 0.014]. The left panels show example traces. 10 ms/100 pA, 10 ms/200 pA, 10 ms/200 pA, 10 ms/50 pA, 10 ms/50 pA, and 10 ms/50 pA for preadolescent female, adolescent female, adult female, preadolescent male, adolescent male, and adult male groups, respectively. Horizontal line in each group represents mean and vertical line represents SEM. **B**) NMDA receptor transmission in IL-mPFC VIPINs. Comparison of NMDA receptor transmission in tdTomato-expressing VIPINs from female preadolescent (n = 6 neurons/3 mice), male preadolescent (n = 7 neurons/3 mice), female adolescent (n = 5 neurons/3 mice), male adolescent (n = 6 neurons/3 mice), female adult (n = 7 neurons/3 mice), and male adult mice (n = 7 neurons/3 mice) have revealed a suppression of NMDA receptor transmission during development from preadolescence to adulthood. A two-way ANOVA of NMDA EPSC amplitude at 9 mA stimulation showed an effect of age [F (2, 37) = 9.9, *P* = 0.001, *P* = 0.005 for preadolescent vs adolescent, *P* = 1 for adult vs adolescent, *P* = 0.001 for adult vs preadolescent]. However, we did not observe an effect of sex [F (1, 37) = 0.55, *P* = 0.47] or an interaction between age and sex [F (2, 37) = 0.85, *P* = 0.44]. We did not observe an effect of age [F (2, 37) = 2.2, *P* = 0.13], an effect of sex [F (1, 37) = 0.13, *P* = 0.72], or an interaction between age and sex [F (2, 37) = 1.9, *P* = 0.16] in NMDA EPSC decay time. The upper panel shows example traces. 25 ms/50 pA.

Our recent study shows that SSTINs but not PVINs exhibit a development-dependent switch in the subunit composition of AMPA receptors (Koppensteiner et al. 2019c). Although both SSTINs and PVINs express synaptic GluA2 subunit-lacking synaptic calcium-permeable AMPA receptors (CPARs), a key mediator of plasticity in GABAergic neurons (Lamsa et al. 2007), these receptors in SSTINs switch to GluA2 subunit-containing AMPA receptors during development (Koppensteiner et al. 2019c). Therefore, we examined whether the AMPA receptor subunit composition undergoes a development-dependent plasticity in VIPINs. Since synaptic CPARs exhibit an inward rectification of EPSCs, we compared the inward rectification of electrically evoked AMPA EPSCs in the tdTomato-expressing VIPINs from preadolescent, adolescent, and adult VIP-tdTomato mice. Unlike PVINs or SSTINs, VIPINs did not show a notable inward rectification, suggesting the presence of GluA2 subunit-containing AMPA receptors in VIPINs (Fig. 3*A*). Furthermore, a comparison of rectification index in the IL-mPFC VIPINs of the preadolescent, adolescent, and adult groups did not show a statistically significant effect of age or sex. However, a statistically significant interaction of age and sex suggests changes in AMPA receptor subunit composition during development in a sex-dependent manner.

NMDA receptors, which play a crucial role in calcium signaling and synaptic plasticity, undergo a development-dependent plasticity in PVINs and SSTINs (Koppensteiner et al. 2019c). Therefore, we asked whether VIPINs show a similar change in NMDA receptor-mediated glutamatergic transmission during development from preadolescence to adulthood. A comparison of the amplitude of electrically evoked NMDA EPSCs in tdTomato-positive VIPINs revealed a development-dependent decrease in NMDA receptor transmission (Fig. 3*B*). The adolescent and adult groups showed a significantly lower NMDA receptor transmission compared with the preadolescent group (Fig. 3*B*). Unlike the NMDA EPSC amplitude, we did not observe any difference in NMDA EPSC decay time, suggesting a lack of subunit-specific changes in NMDA receptors during development (Fig. 3*B*).

### GABAergic transmission in IL-mPFC VIPINs in preadolescent, adolescent, and adult mice

**Figure 4:**
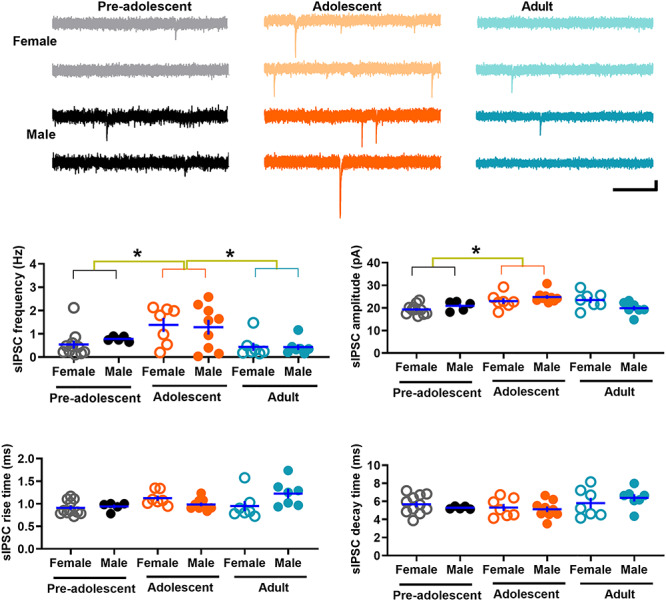
Spontaneous GABAergic transmission in IL-mPFC VIPINs. Frequency, amplitude, rise time, and decay time of sIPSCs in tdTomato-expressing VIPINs from female preadolescent (n = 11 neurons/3 mice), male preadolescent (n = 5 neurons/3 mice), female adolescent (n = 7 neurons/3 mice), male adolescent (n = 9 neurons/3 mice), female adult (n = 7 neurons/3 mice), and male adult mice (n = 7 neurons/3 mice). Horizontal line in each group represents mean and vertical line represents SEM. The upper panels show example traces. Scale, 250 ms/10 pA. A two-way ANOVA of sIPSC frequency revealed an effect of age [F (2, 45) = 8.12, *P* = 0.001, *P* = 0.009 for preadolescent vs adolescent, *P* = 0.001 for adult vs adolescent, *P* = 1 for adult vs preadolescent] but no effect of sex [F (1, 45) = 0.05, *P* = 0.82] or an interaction between age and sex [F (2, 45) = 0.28, *P* = 0.76]. We also observed an effect of age [F (2, 45) = 7.1, *P* = 0.002, *P* = 0.001 for preadolescent vs adolescent, *P* = 0.07 for adult vs adolescent, *P* = 0.22 for adult vs preadolescent] and an interaction between sex and age [F (2, 45) = 4.6, *P* = 0.02] in sIPSC amplitude. However, we did not observe an effect of sex [F (1, 45) = 0.004, *P* = 0.95]. An analysis of sIPSC rise time did not reveal an effect of age [F (2, 45) = 2.6, *P* = 0.085] or sex [F (1, 45) = 0.85, *P* = 0.36]. However, we observed an interaction between sex and age [F (2, 45) = 4.1, *P* = 0.023] in sIPSC rise time. A two-way ANOVA of sIPSC decay time did not reveal an effect of age [F (2, 45) = 2.6, *P* = 0.09], an effect of sex [F (1, 45) = 0.00, *P* = 1], or an interaction between age and sex [F (2, 45) = 0.8, *P* = 0.45].

An alteration in the GABAergic drive onto VIPINs could affect their synaptic output. To study GABAergic transmission in VIPINs, we examined spontaneous GABAergic transmission in tdTomato-expressing VIPINs from preadolescent, adolescent, and adult mice by comparing the frequency, amplitude, rise time, and decay time of sIPSCs (Fig. 4). The sIPSC frequency in the adolescent group was significantly higher than that in both the preadolescent and adult groups (Fig. 4). Although the sIPSC amplitude in the adolescent group was higher than that in both the preadolescent and adult groups, only the preadolescent vs adolescent comparison showed a statistical significance (Fig. 4). Thus, VIPINs in the IL-mPFC exhibit an elevated GABAergic input during adolescence. Also, we observed an interaction of age and sex on sIPSC amplitude and rise time, suggesting potential sex-dependent changes in the subunit composition of GABA_A_ receptors during development.

### VIPIN-pyramidal neuron GABAergic transmission in preadolescent, adolescent, and adult mice

**Figure 5:**
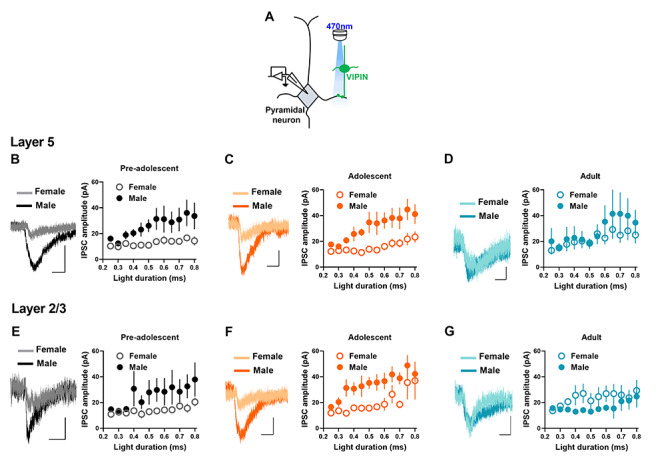
VIPIN-pyramidal neuron synaptic transmission in the IL-mPFC. **A**) Schematic of optogenetic measurement of VIPIN-pyramidal neuron synaptic transmission. **B–D**) VIPIN-mediated currents in the IL-mPFC layer 5 pyramidal neurons of VIP-ChR2 mice (preadolescent group: n = 14 neurons/5 male mice, n = 15 neurons/5 female mice, adolescent group: n = 14 neurons/5 male mice, n = 16 neurons/5 female mice, adult group: n = 14 neurons/5 male mice, n = 15 neurons/5 female mice). A two-way ANOVA of IPSC amplitude at 0.5 ms light stimulation did not show an effect of age [F (2, 87) = 1.14, *P* = 0.32]. However, we observed an effect of sex [F (1, 87) = 10.5, *P* = 0.002]. The interaction between age and sex was not significant [F (2, 87) = 3.1, *P* = 0.051]. Example traces (0.5 ms light duration) are shown on the left. Scale, 10 ms/50 pA (preadolescent), 10 ms/20 pA (adolescent), and 10 ms/10 pA (adult). **E–G**) VIPIN-mediated currents in the IL-mPFC layer 2/3 pyramidal neurons of VIP-ChR2 mice (preadolescent group: 14 = neurons/5 male mice, 16 = neurons/5 female mice, adolescent group: n = 17 neurons/5 male mice, n = 17 neurons/5 female mice, adult group: n = 13 neurons/5 male mice, n = 15 neurons/5 female mice). A two-way ANOVA of IPSC amplitude at 0.5 ms light stimulation did not show an effect of age [F (2, 91) = 1.4, *P* = 0.26]. However, we observed an effect of sex [F (1, 91) = 4.4, *P* = 0.03] and an interaction between age and sex [F (2, 91) = 4.2, *P* = 0.018]. Example traces (0.5 ms light duration) are shown on the left. Scale, 10 ms/20 pA (preadolescent), 10 ms/20 pA (adolescent), and 10 ms/20 pA (adult).

Next, we examined VIPIN-mediated GABAergic transmission in pyramidal neurons during development from preadolescence to adulthood. We measured the amplitude of light-evoked inhibitory postsynaptic currents (IPSCs) in the IL-mPFC layer 5 and layer 2/3 pyramidal neurons from preadolescent, adolescent, and adult VIP-ChR2 mice. Since neurons that express VIP and choline acetyltransferase release acetylcholine in the mPFC (Obermayer et al. 2019), we have confirmed the GABAergic nature of VIPIN-pyramidal neuron transmission by blocking light-evoked currents with bicuculline, a GABA_A_ receptor blocker. Bicuculline completely blocked light-evoked responses in layer 5 pyramidal neurons (Supplementary Fig. 2). We undertook a comparison of VIPIN-mediated GABAergic transmission in layer 5 pyramidal neurons in female and male preadolescent, adolescent, and adult mice. A statistical comparison did not reveal a development-dependent change in IPSC amplitude or an interaction between development and sex (Fig. 5*B–D*). However, we observed an effect of sex on IPSC amplitude. We made a similar analysis of VIPIN-mediated GABAergic transmission in layer 2/3 pyramidal neurons in the preadolescent, adolescent, and adult groups (Fig. 5*E–G*). Although a statistical comparison did not reveal a development-dependent change in IPSC amplitude, we observed an effect of sex and an interaction between sex and development on VIPIN-mediated GABAergic transmission in layer 2/3 pyramidal neurons (Fig. 5*B–D*). Overall, these data show sex difference in VIPIN-mediated inhibition of pyramidal neurons in the IL-mPFC. Also, the magnitude of IPSC amplitude in all 3 developmental stages suggests a minimal VIPIN-mediated inhibition of pyramidal neurons in the IL-mPFC.

### Neurite density, soma area, and density of VIPINs in the IL-mPFC of preadolescent, adolescent, and adult mice

Since VIPINs exhibited development- and sex-specific changes in synaptic and membrane properties, we examined whether this prolonged developmental period from preadolescence to adulthood also involves morphological changes in VIPINs. First, we examined the neurite density of VIPINs in preadolescent, adolescent, and adult mice by comparing fluorescence intensity of tdTomato-expressing neurites in VIP-tdTomato mice. Although these experiments revealed a development-dependent increase in neurite density (Supplementary Fig. 3), we did not observe any sex difference in this effect or an interaction between sex and age. Similarly, we observed a development-dependent increase in the soma area (Supplementary Fig. 4), suggesting that the morphological changes in the IL-mPFC VIPINs are prolonged. Similar to neurite density, we did not observe a sex-specific effect in soma area or an interaction between sex and age. However, we observed a development-dependent decrease in the density of VIPINs without an effect of sex or an interaction between sex and age (Supplementary Fig. 5).

## Discussion

A notable observation from this study is the enhanced synaptic inhibition of VIPINs during adolescence. Furthermore, the membrane excitability of VIPINs is diminished during adolescence. Since the VIPINs play a critical role in disinhibition due to their preferential innervation of other GABAergic neurons (Lee et al. 2013; Pfeffer et al. 2013; Pi et al. 2013; Adler et al. 2019; Krabbe et al. 2019), both the enhanced GABAergic inputs and a diminished membrane excitability in VIPINs during adolescence could affect their role in mediating cortical disinhibition. Our earlier studies have demonstrated an attenuation of both glutamatergic and intrinsic plasticity in the IL-mPFC pyramidal neurons during adolescence (Pattwell et al. 2012; Koppensteiner et al. 2019b). Since SSTINs are a major synaptic target of VIPINs (Lee et al. 2013; Pfeffer et al. 2013; Pi et al. 2013), a diminished excitation of VIPINs could allow for an enhanced SSTIN-mediated inhibition of pyramidal neurons. We have recently observed an enhanced SSTIN-pyramidal neuron GABAergic transmission in the IL-mPFC during adolescence (Koppensteiner et al. 2019c). Therefore, a decrease in the VIPIN-mediated inhibition of SSTINs could diminish the disinhibition of pyramidal neurons and hence, their ability to undergo plasticity during adolescence. Although the mechanism underlying increased GABAergic transmission and diminished excitability in VIPINs during adolescence is unclear, neuromodulation mediated by serotonin or dopamine could play a role in adolescence-specific changes in VIPINs. VIPINs but not PVINs or SSTINs express both 5-HT3 and D1 receptors (Goldman-Rakic et al. 2004; Spencer et al. 2015; Whittle et al. 2016; Anastasiades et al. 2019; Hare et al. 2019). Importantly, both the 5-HT3 and dopamine D1 receptors are important for threat extinction, which is diminished during adolescence (Park and Williams 2012; Kondo et al. 2013; Whittle et al. 2016). The diminished threat extinction in adolescents is associated with a downregulation of D1 receptors in the IL-mPFC (Zbukvic et al. 2017). Also, the density of 5-HT expressing axonal fibers in the mPFC is suppressed during adolescence, which is consistent with the previous studies in the primary sensory cortex (D'Amato et al. 1987; Dori et al. 1996; Maddaloni et al. 2017). Therefore, adolescence-specific changes in serotonergic or dopaminergic transmission could play an important role in the enhanced GABAergic transmission and diminished membrane excitability in VIPINs.

Another significant finding from our study is the sex difference in VIPIN-mediated GABAergic transmission in pyramidal neurons. As reported earlier in other cortical areas, the VIPIN-mediated inhibition of pyramidal neurons in the IL-mPFC is minimal (Pfeffer et al. 2013; Pi et al. 2013). VIPINs primarily target other GABAergic neurons, particularly SSTINs, to mediate a disinhibitory effect (Lee et al. 2013; Pfeffer et al. 2013; Pi et al. 2013; Krabbe et al. 2019). Since sex hormones exert a robust effect on GABAergic synapses (Murphy et al. 1998), the observed sex difference in VIPIN-pyramidal neuron transmission could involve sex hormone-dependent effects. However, it is also possible that sex hormone-independent effects might contribute to this sex difference. Cortical VIPINs show an exclusive expression of bombesin BB2 receptors, which are the primary target of gastrin releasing peptide (Jensen et al. 2008; Huntley et al. 2020). Interestingly, the gene encoding BB2 receptors is located on the X chromosome and studies show that this gene escapes X inactivation (Maslen and Boyd 1993; Ishikawa-Brush et al. 1997), suggesting its potential role in sex differences. Furthermore, an altered BB2 receptor signaling is involved in diminished threat extinction (Martel et al. 2012). Despite the diminished excitability and an increased inhibition of VIPINs during adolescence, the VIPIN-pyramidal neuron GABAergic transmission did not exhibit an adolescence-specific decrease, which might be due to an early maturation postsynaptic GABAergic mechanisms in the pyramidal neurons (Koppensteiner et al. 2019b). Although our imaging experiments showed a development-dependent increase in VIPIN neurite density and soma area in the IL-mPFC, we did not observe any sex difference in these effects. Also, the VIPIN density decreased during development in a sex-independent manner. Therefore, it is possible that the sex-specific changes in VIPINs are limited to synaptic transmission without affecting either the membrane properties or morphology.

Unlike the GABAergic transmission in VIPINs, the strength of AMPA receptor-mediated glutamatergic transmission remains stable during development from preadolescence to adulthood. In contrast to SSTINs and PVINs, which show the presence of GluA2 subunit-lacking CPARs (Koppensteiner et al. 2019a), VIPINs do not exhibit a robust presence of synaptic CPARs. Interestingly, we observed an interaction between development and sex in both sEPSC kinetics and inward rectification of AMPA currents suggesting dynamic sex-specific changes in AMPA receptor transmission during development. Unlike the AMPA receptor transmission, the NMDA receptor transmission is attenuated in VIPINs after the preadolescence period. Given the diminished presence of calcium permeable AMPA receptors and NMDA receptors, 2 major sources of synaptic calcium signaling, it is unclear whether VIPINs undergo the classical Hebbian plasticity mediated by NMDA receptors or the anti-Hebbian plasticity mediated by calcium permeable AMPA receptors (Lamsa et al. 2005; Lamsa et al. 2007).

Our results also show that active membrane properties in VIPINs are extremely sensitive to temperature changes, as an increase in temperature suppressed the number of evoked action potentials. Previous studies have shown a similar effect of temperature on neuronal excitability (Van Hook 2020). The mechanism underlying the effect of temperature on active membrane properties could involve temperature-dependent changes in neurotransmitter or neuropeptide release, membrane currents, metabolic rate, gas solubility, availability of oxygen, or thermosensitive channels (Shibasaki et al. 2007; de la Peña et al. 2012; Kim and Connors 2012). However, we did not observe a significant change in passive membrane properties. In contrast to our findings, previous studies have also shown an increase in excitability in hippocampal neurons in response to hyperthermia (Kim and Connors 2012). Consistent with an increase in input resistance during development, we observed a decrease in the membrane capacitance in adult group compared with both the preadolescent and adolescent mice. However, a development-dependent increase in both neurite density and soma area is not consistent with these changes in passive membrane properties, suggesting the need for advanced microscopy studies to understand development-dependent membrane remodeling in VIPINs. Recent studies do not show evidence for robust myelination in VIPINs (Micheva et al. 2016; Zonouzi et al. 2019). Therefore, other development-dependent modifications in the membrane might be responsible for an increased input resistance and decreased membrane capacitance in adult VIPINs.

In conclusion, our results show an adolescence-specific plasticity in the IL-mPFC VIPINs characterized by an increase in GABAergic transmission and a decrease in membrane excitability. This increased synaptic inhibition of VIPINs and the decrease in VIPIN excitability might result in a diminished disinhibition in the IL-mPFC pyramidal neurons, activity of which is necessary for the expression of threat extinction. Therefore, the adolescence-specific VIPIN plasticity might play a role in the reduced threat extinction during adolescence. Furthermore, the sex difference in the GABAergic output from VIPINs might play a role in sex difference in anxiety disorders during adolescence.

## Funding

This work was supported by the National Institutes of Health (grant number HD076914 to I.N.).

## Notes

Authors thank the laboratory of Dr Robert E McCullumsmith and Dr Andrea Kalinoski (Integrated Core Facilities) for help with the experiments. *Conflict of Interest*: None declared.

## Supplementary Material

Supplementary_Figures_tgab007
