# Supplementary material for: Development-Dependent Plasticity in Vasoactive Intestinal Polypeptide Neurons in the Infralimbic Cortex
Source: Cereb Cortex Commun. 2021 Feb 4;2(1):tgab007. doi: 10.1093/texcom/tgab007 (PMC7948133; doi:10.1093/texcom/tgab007)
Supplement: Supplementary_Figures_tgab007 [file supplementary_figures_tgab007.pdf]

Supplementary Figures

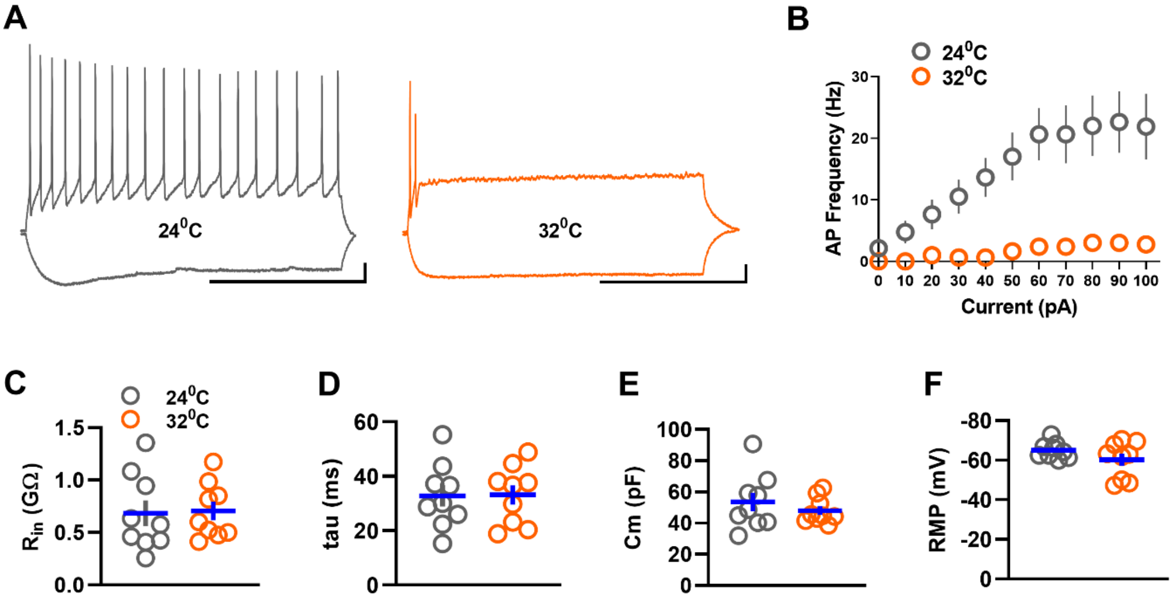

S. Figure 1 Effect of temperature on membrane properties of VIPINs. **A**) Example traces of voltage responses to hyperpolarizing (-50 pA) and depolarizing (+100 pA) current steps in tdTomato expressing VIPINs from VIP-tdTomato mice. Scale = 500 ms/10 mV. **B**) Mean action potential (AP) frequency in response to current injection in VIPINs from the 24^0C (n=9 neurons/3 mice) and the 32^0C (n=9 neurons/3 mice) groups (P29-P31). Action potential frequency at higher temperature (32^0C) was significantly lower than that at lower temperature (24^0C) [$P < 0.001$ at 100 pA current injection]. However, temperature did not affect input resistance (Rin, **C**), membrane time constant (tau, **D**), membrane capacitance (Cm, **E**), or resting membrane potential (RMP, **F**). Horizontal line in each group represents mean and vertical line represents SEM.

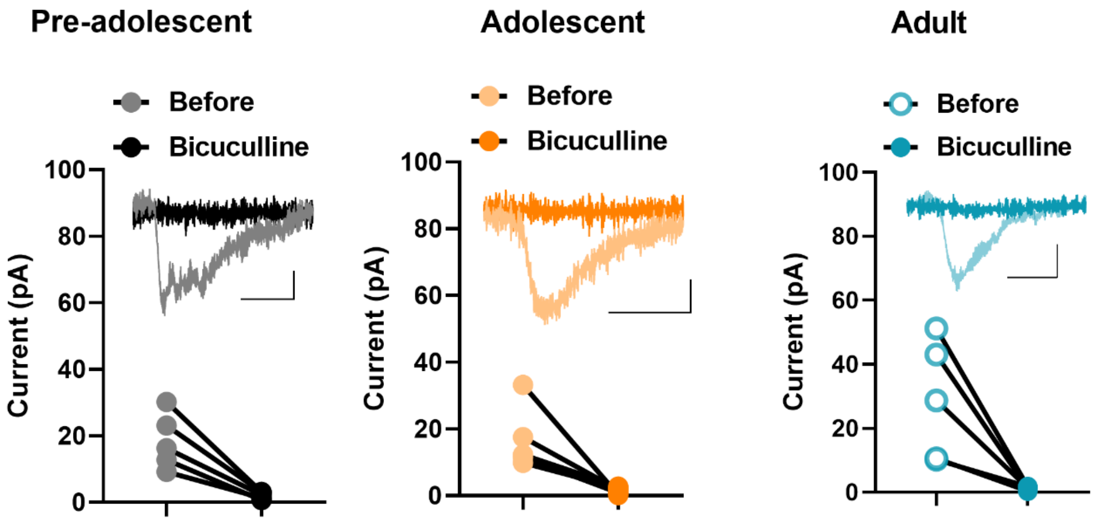

S. Figure 2 Confirmation of VIPIN-mediated GABAergic transmission in pyramidal neurons. The GABAergic nature of VIPIN-mediated synaptic transmission in layer 5 pyramidal neurons from pre-adolescent (5 neurons/2 male mice), adolescent (4 neurons/2 male mice) and adult (4 neurons/2 male mice) VIP-ChR2 mice was confirmed by blocking the light-evoked currents with $GABA_A$ receptor blocker bicuculline.

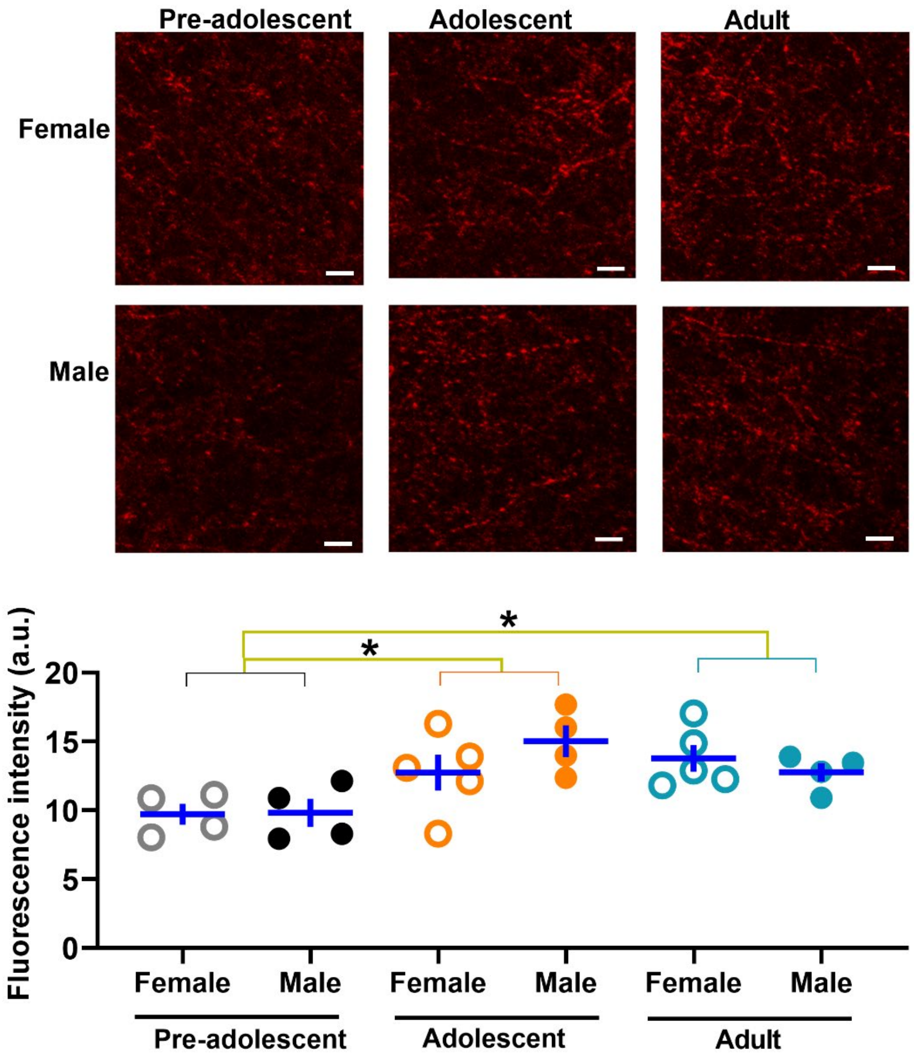

S. Figure 3 VIPIN neurite density in the IL-mPFC measured by comparing fluorescence intensity of tdTomato-expressing neurites. Comparison of pre-adolescent [n=8 (4 female mice+4 male mice)], adolescent [n=9 (5 female mice+4 male mice)], and adult [n=9 (5 female mice+4 male mice)] groups revealed an effect of age [$F(2, 25) = 8.7$, $P = 0.002$, $P = 0.003$ for pre-adolescent vs adolescent, $P = 1$ for adult vs adolescent, $P = 0.009$ for adult vs pre-adolescent] but no effect of sex [$F(1, 25) = 0.3$, $P = 0.6$] or an interaction between sex and age [$F(2, 25) = 1.3$, $P = 0.3$]. Horizontal line in each group represents mean and vertical line represents SEM. Upper panel shows example images. Scale 15 μm.

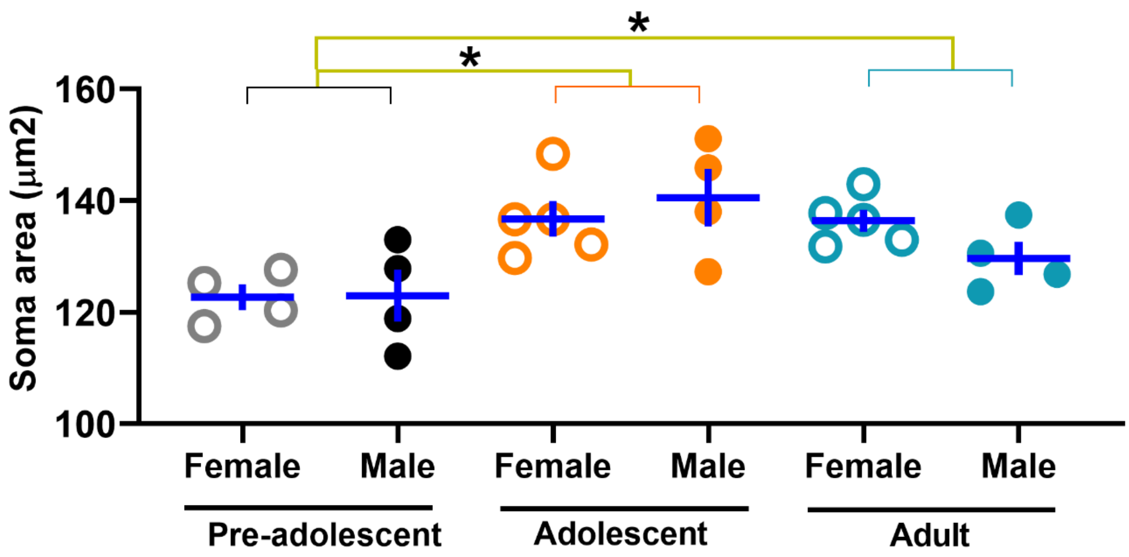

S. Figure 4 VIPIN soma area in the IL-mPFC. Comparison of pre-adolescent [n=8 (4 female mice+4 male mice)], adolescent [n=9 (5 female mice+4 male mice)], and adult [n=9 (5 female mice+4 male mice)] groups revealed an effect of group [F (2, 25) = 10.39, P = 0.001, P = 0.001 for pre-adolescent vs adolescent, P = 0.46 for adult vs adolescent, P = 0.02 for adult vs pre-adolescent] without an effect of sex [F (1, 25) = 0.1, P = 0.75] or an interaction between sex and age [F (2, 25) = 1.24, P = 0.3]. Horizontal line in each group represents mean and vertical line represents SEM.

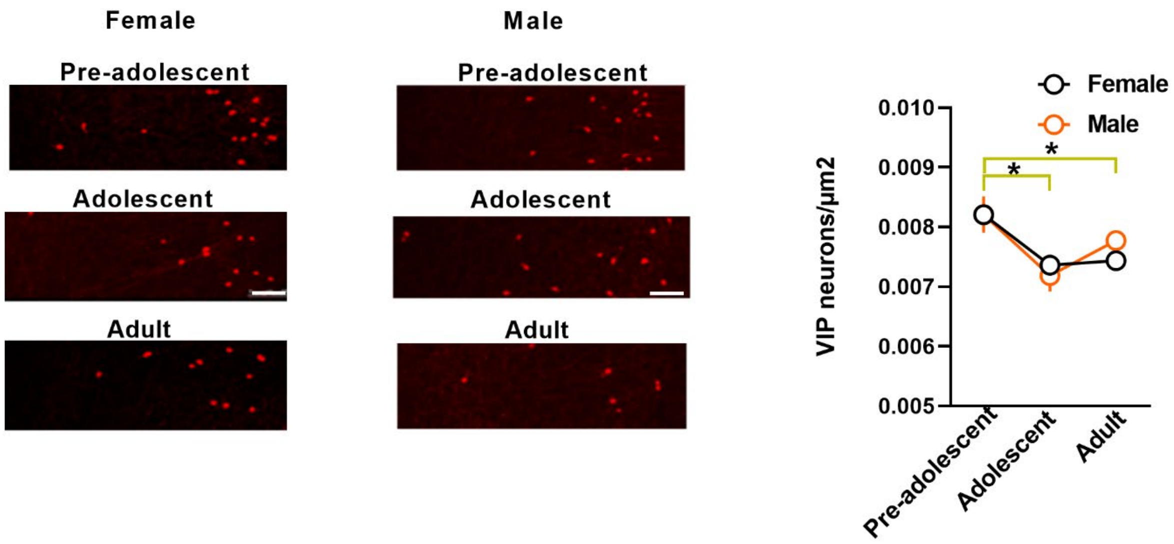

S. Figure 5 VIPIN density in the IL-mPFC of pre-adolescent (4 female mice+4 male mice), adolescent (5 female mice+4 male mice), and adult (5 female mice+4 male mice)] groups. A two-way ANOVA revealed an effect of age on density of tdTomato neurons [F (2, 25) = 10.9, P = 0.001, P = 0.001 for pre-adolescent vs adolescent, P = 0.41 for adult vs adolescent, P = 0.018 for adult vs pre-adolescent], without an effect of sex [F (1, 25) = 0.11, P = 0.74] or an interaction between sex and age [F (2, 25) = 0.88, P = 0.43]. Left panel shows example images. Scale 100 µm.
